# Global trends in research on miRNA–microbiome interaction from 2011 to 2021: A bibliometric analysis

**DOI:** 10.3389/fphar.2022.974741

**Published:** 2022-08-30

**Authors:** Xiang-Yun Yan, Jun-Peng Yao, Yan-Qiu Li, Wei Zhang, Meng-Han Xi, Min Chen, Ying Li

**Affiliations:** ^1^ The Third Hospital/Acupuncture and Tuina School, Chengdu University of Traditional Chinese Medicine, Chengdu, China; ^2^ Academic Affairs Office, Chengdu University of Traditional Chinese Medicine, Chengdu, China; ^3^ Clinical Medicine School, Hospital of Chengdu University of Traditional Chinese Medicine, Chengdu, China

**Keywords:** miRNA-microbiome, interaction, bibliometrix, VOSviewer, bibliometrics

## Abstract

An increasing number of research suggests that the microRNA (miRNA)–microbiome interaction plays an essential role in host health and diseases. This bibliometric analysis aimed to identify the status of global scientific output, research hotspots, and frontiers regarding the study of miRNA–microbiome interaction over the past decade. We retrieved miRNA–microbiome-related studies published from 2011 to 2021 from the Web of Science Core Collection database; the R package bibliometrix was used to analyze bibliometric indicators, and VOSviewer was used to visualize the field status, hotspots, and research trends of miRNA–microbiome interplay. In total, 590 articles and reviews were collected. A visual analysis of the results showed that significant increase in the number of publications over time. China produced the most papers, and the United States contributed the highest number of citations. Shanghai Jiaotong University and the University of California Davis were the most active institutions in the field. Most publications were published in the areas of biochemistry and molecular biology. Yu Aiming was the most prolific writer, as indicated by the h-index and m-index, and Liu Shirong was the most commonly co-cited author. A paper published in the International Journal of Molecular Sciences in 2017 had the highest number of citations. The keywords “expression” and “gut microbiota” appeared most frequently, and the top three groups of diseases that appeared among keywords were cancer (colorectal, et al.), inflammatory bowel disease (Crohn’s disease and ulcerative colitis), and neurological disorders (anxiety, Parkinson’s disease, et al.). This bibliometric study revealed that most studies have focused on miRNAs (e.g., miR-21, miR-155, and miR-146a), gut microbes (e.g., *Escherichia coli*, *Bifidobacterium*, and *Fusobacterium nucleatum*), and gut bacteria metabolites (e.g., butyric acid), which have the potential to improve the diagnosis, treatment, and prognosis of diseases. We found that therapeutic strategies targeting the miRNA–microbiome axis focus on miRNA drugs produced *in vitro*; however, some studies suggest that *in vivo* fermentation can greatly increase the stability and reduce the degradation of miRNA. Therefore, this method is worthy of further research.

## 1 Introduction

Approximately 100 trillion microbes inhabit the human body (Peter et al., 2007), and nearly 95% of these microbes colonize the gastrointestinal tract. These microbes comprise a wide variety of species, including bacteria, viruses, parasites, and fungi (Kathryn and James, 2012). The microbiome plays a critical role in human health, mediating the host’s physiological functions related to nutrition, barrier function, and immunity (Dai et al., 2015; Feng et al., 2018; Pang et al., 2018; Viggiano et al., 2015). Because microbiomes are very adaptable, they are susceptible to many host factors, including genetics, age, diet, stress, and disease conditions (Horne et al., 2019; Miro-Blanch and Yanes, 2019). When microbiome dysbiosis occurs, the biological balance is broken, provoking host inflammation, immune disorders, metabolic disorders, and other pathological conditions (James et al., 2011; Xian-Qian et al., 2018). Recently, more and more studies have focused on the mechanisms by which the microbiome influences human health and disease, suggesting that microRNA (miRNA) could play a critical role in human–microbiome interactions.

miRNAs are small non-coding RNAs about 22 nucleotides in length that regulate target gene expression (David, 2004). First discovered in nematodes, they have since been identified in many forms of life, including viruses, plants, and animals (Horvitz and Sulston, 1980; Peter et al., 2008; Weronika et al., 2022). By binding the 3′ UTR of target genes, miRNAs can inhibit the process of mRNA translation or accelerate mRNA degradation, ultimately regulating cell development, proliferation, and apoptosis (Marc and Nahum, 2012; TX and ME, 2018). Numerous studies have shown that miRNAs have a role in the onset and progression of illness, and miRNAs have been identified as diagnostic biomarkers and therapeutic targets for a wide range of disorders (Rashid et al., 2020; Pavithra et al., 2021). Studies in the past several years have uncovered interactions between miRNAs and the microbiome that modulate host health and diseases.

In 2011, Dalmasso et al. (2011) discovered nine miRNAs differently expressed in the ileum and the colon from colonized mice that were lacking in germ-free mice. Interestingly, subsequent research has shown that miRNAs generated from dietary supplements may stimulate host gene expression, hence influencing the host–microbiota interplay; however, few are revealed about this process’s mechanism (Gupta et al., 2015; Yi and Kim, 2021). In 2016, Liu et al. (2016) demonstrated that host fecal miRNAs may be able to alter bacterial composition by targeting bacterial genes selectively; this finding was a crucial milestone in understanding the mechanism of the interaction between the microbiome and miRNA. Dysregulation of the symbiosis between the miRNA and the microbiota is linked with a range of diseaseas, which include inflammatory bowel disease (IBD), colon cancer, and neurological disorders (Ragusa et al., 2020; Cao et al., 2021; Casado-Bedmar and Viennois, 2021). Recently, miRNA–microbiome interplay has become a research hotspot and the focus of many reviews (Jiayi et al., 2019; Deepansh et al., 2021), but comprehensive and visual reviews in this field are limited.

Bibliometric analysis is a statistical method that can be used for both qualitative and quantitative analysis and the evaluation of emerging trends in scientific research (Xiuqing et al., 2021). Despite the rapid growth in miRNA–microbiome interaction research over the past decade, no bibliometric analysis has been published in this field. Using the Web of Science Core Collection (WoSCC) database, we identified miRNA–microbiome-related studies published from 2011 to 2021. We used the R package bibliometrix and VOSviewer to visualize the global research status, hotspots, and trends in the area of miRNA–microbiome interaction. Furthermore, we searched for potential diagnostic biomarkers and therapies for various diseases, aiming to provide insight into accessible clinical applications of miRNA–microbiome interplay.

## 2 Methods

### 2.1 Data source and search strategy

All of the data were obtained from the WoSCC database, which includes high-quality scholarly peer-reviewed literature published worldwide and commonly used in bibliometrics (Mathew et al., 2008). Considering database renewal, literature retrieval was conducted on 6 March 2022. The search terms were “miRNA,” “microRNA,” “miRNAs,” “microbiome,” “microbial,” “microflora,” “microbiota,” “probiotic,” “microorganism,” “*Saccharomyces*,” “*Lactobacillus*,”, “*Bifidobacterium*,” and “*E. coli*”. The present analysis was concerned with articles and reviews from 2011 to 2021. Publications irrelevant to the search subject were omitted. After the removal of duplicates, a text file containing the remaining 590 analyzed records was downloaded.

### 2.2 Bibliometric analysis and visualization

In our study, the distribution of countries/regions, years of publication, and authors were analyzed by using the bibliometrix package in R (version 4.1.2). The fitting polynomial model was employed to better demonstrate the variations in the yearly document quantity. Meanwhile, we used the following indicators to assess publication quality by authors: Publications, citations, h-index (H), and m-index (M) are used to assess a researcher’s academic contribution and forecast future scientific breakthroughs (Hirsch, 2005). To evaluate the quality of Journals, we also obtained the 2021 impact factor (IF) and JCR division of journals from the WoSCC, which are often regarded as one of the most important indices of the quality and influence of medical journals (Ernesto et al., 2019).

The VOSviewer Version 1.6.18 (Centre for Science and Technology Studies, Leiden University, Leiden, Netherlands) was utilized to map bibliometrics, such as co-authorship analysis of authors/institutions/countries, citation analysis of documents, co-citation analysis of journals/references, and co-occurrence analysis of keywords. The following are the VOSviewer parameters: counting method is full counting; the minimum number of citations per source is 100; visualization weights is citations; normalization is association strength; resolution for clustering is 1.00; the minimum cluster size is one; minimum line strength is 200; and the maximum number of lines is 500. Moreover, keywords plus that occurred more than five times were shown in two visualizations (network, and density) of the co-occurrence analysis in order to discover hot topics in the interplay of microRNA and microbiome.

## 3 Result

### 3.1 The trends in global publications

**FIGURE 1:**
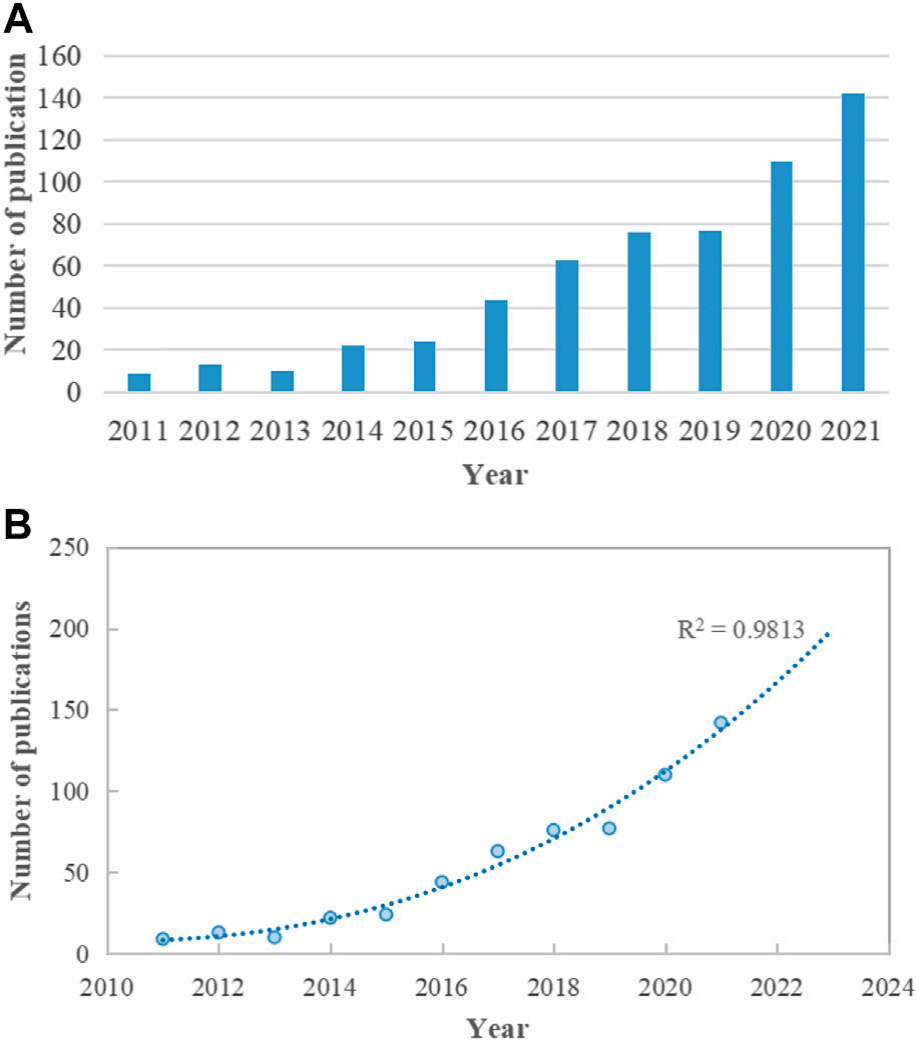
Trends of global publications on the topic of miRNA-microbiome interaction. **(A)** Annual publication output from 2011 to 2021. **(B)** Logistic regression model of growth trends in publications.

WoSCC was searched for 590 articles relating to the relationship between microRNA and microbiome published between 2011 and 2021. From nine publications (1.53%) in 2011 to 142 publications (24.07%) in 2021, the field’s global publications indicated a robust growth tendency. (Figure 1A). The logistic regression model created a time curve indicating that the discipline is now seeing a large increase in the number of yearly publications. (*R* ^2^ = 0.9813) (Figure 1B).

### 3.2 Distribution of countries and institutions

**FIGURE 2:**
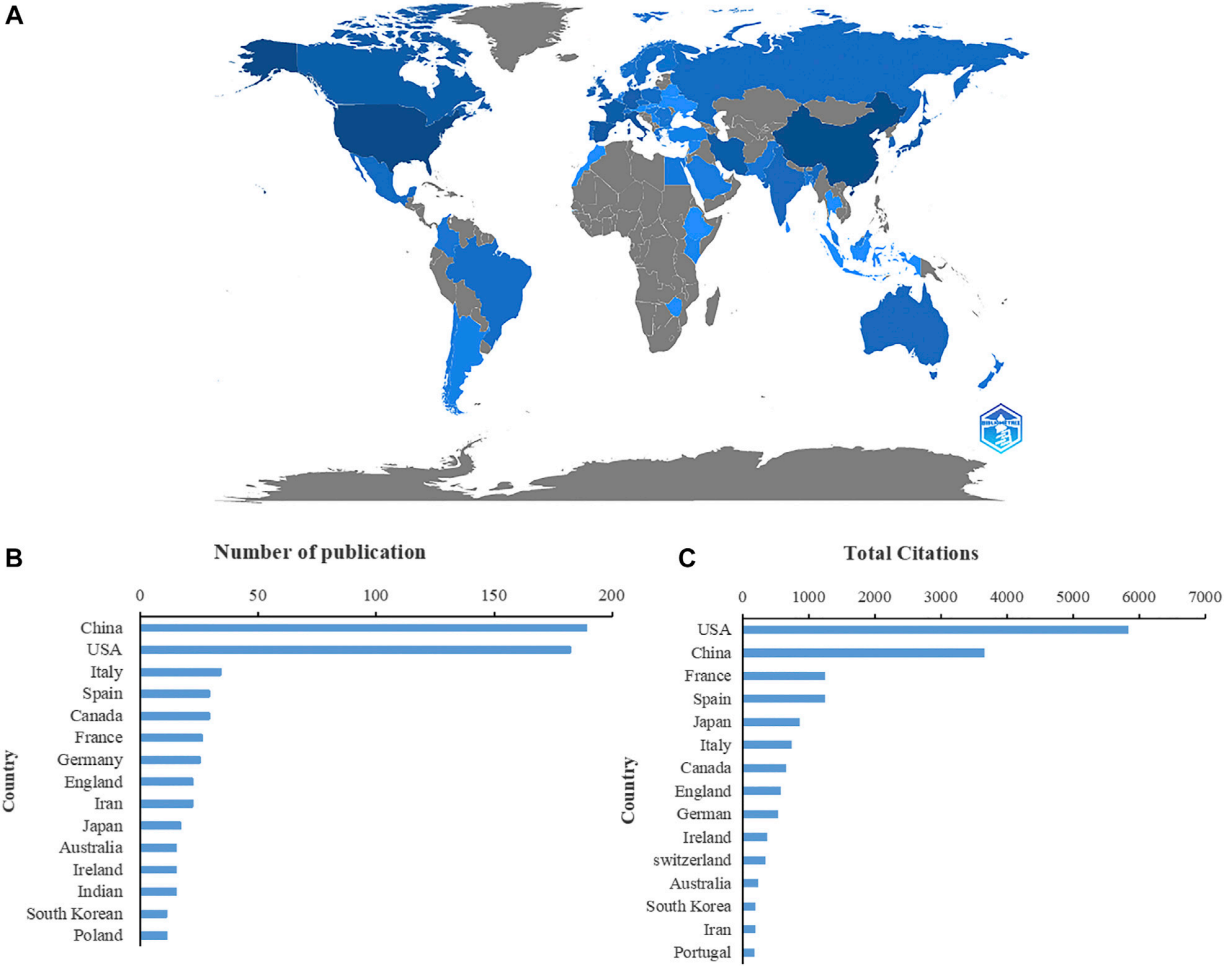
Countries contributing to the study of miRNA-microbiome interaction. **(A)** Distribution of countries in this area on a global map. The darker the blue, the more the number of documents produced by the country. **(B)** The number of publications of the top 15 countries. **(C)** Total number of citations of relevant papers from the 15 leading countries.

**FIGURE 3:**
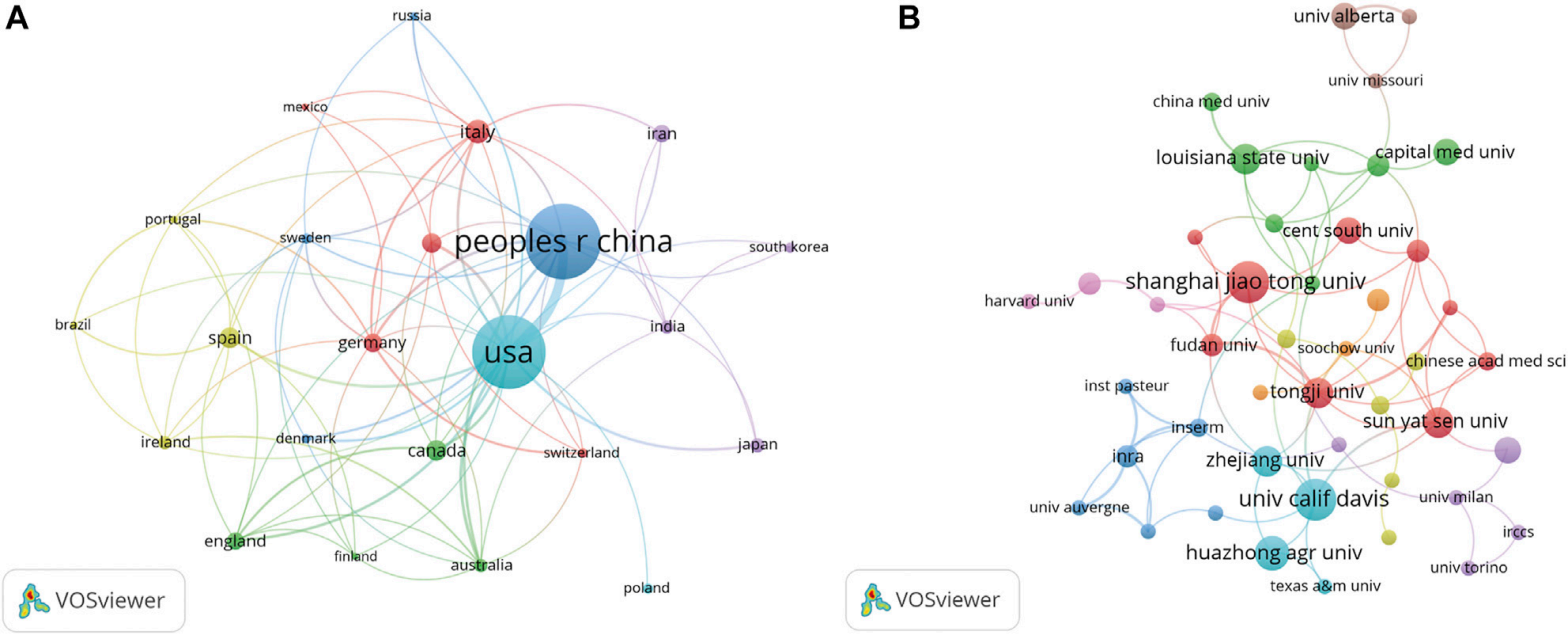
VOSviewer’s network analysis of country and institution co-authorship. The width of the lines represents the strength of the connection. **(A)** The co-authorship analysis of countries with more than five publications. **(B)** The co-authorship analysis of institutions with more than four publications.

The contributions of countries to microRNA and microbiome research were assessed and illustrated by Bibliometric in a globe map (Figure 2A). 49 countries and regions in total contributed to publications in this topic. China contributed the greatest number of publications (189, 32.03%), followed by the United States (182, 30.85%), Italy (34, 5.76%), Spain (29, 4.92%), and Canada (29, 4.92%) (Figure 2B). United States studies received the most citations (5,838), followed by those from China (3,662 citations), France (1,253 citations), Spain (1,251 citations), and Japan (860 citations) (Figure 2C). In the co-authorship analysis, 23 countries with more than five publications in the topic were shown (Figure 3A). The United States topped the list of five countries with the highest total link strength (92), followed by China (70), Italy (19), Germany (17), and Australia (13).

There were a total of 1,009 institutions engaged in this area. Shanghai Jiaotong University and University calif Davis contributed the greatest number of publications (11, 1.86%), followed by Huazhong agriculture University (9, 1.53%), Tongji University, Sun yat-sen University, Zhejiang University, and Louisiana state University all followed with eight publications. We examined the co-authorship of 60 institutions with over four publications. The exclusion of fifteen items that were not connected revealed the collaborations of 45 institutions (Figure 3B). Tongji University ranked first among the top five institutions with the strengthest total link (8), followed by Shanghai Jiaotong University (7), Sun yat-sen University (7), Fudan University (7), and Chinese Academy of Science (6).

### 3.3 Analysis of journals and research areas

**FIGURE 4:**
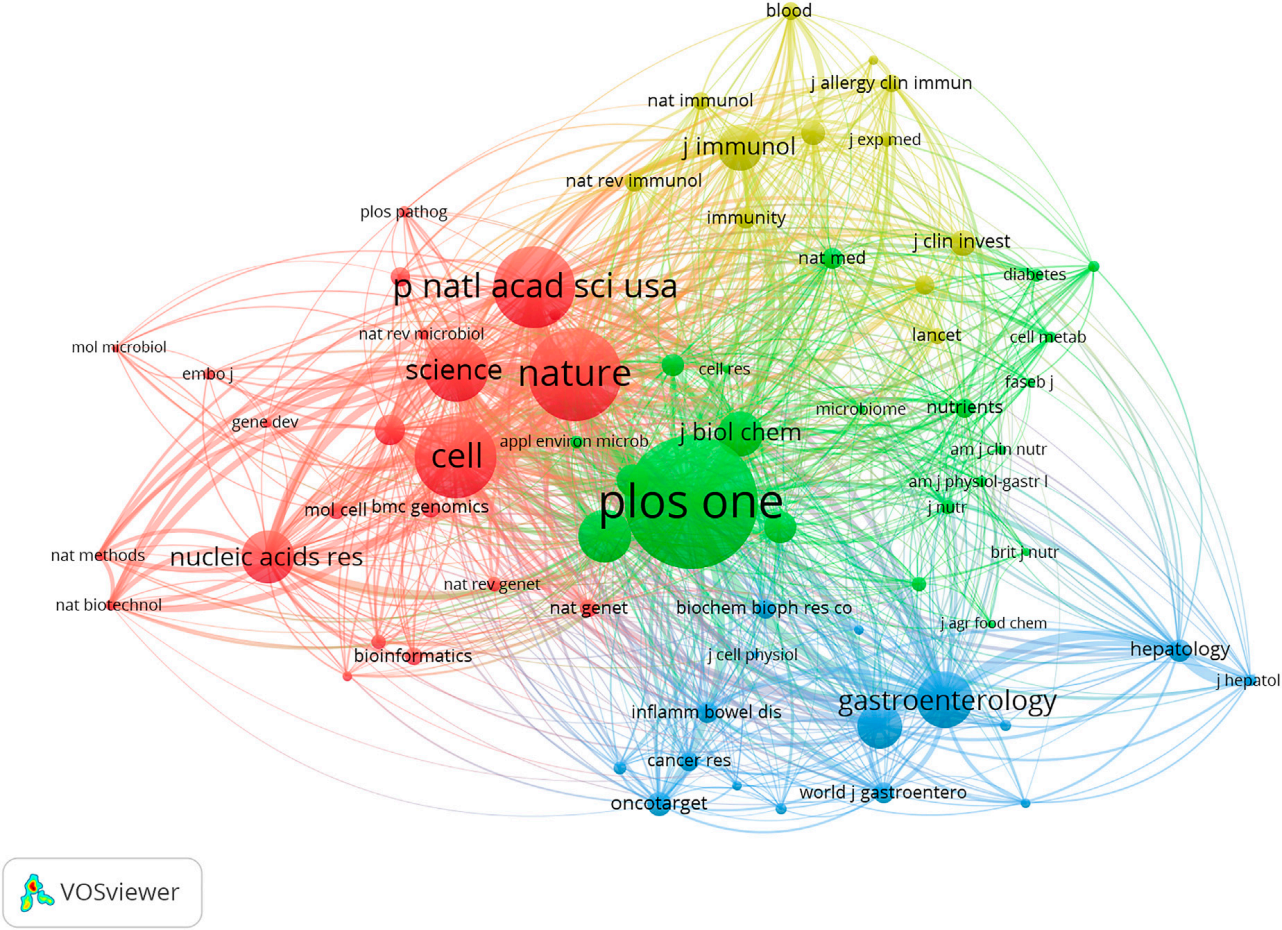
VOSviewer’s network analysis of co-cited journals with more than 50 publications. The node size represents the number of journal papers. The larger the node, the more journal papers.

590 articles altogether were published in 336 journals. Table 1 lists the 10 most popular journals for studies on microRNA and microbiome. *International Journal of Molecular Sciences* (20 records, 3.39%) had the most publications, followed by *Frontiers in Microbiology* (15, 2.54%), *Scientific Reports* (13, 2.20%), *Plos One* (10, 1.69%), and *Frontiers in Immunology* (10, 1.69%). We investigated 71 journals for all papers co-cited in more than 100 publications (Figure 4). Table 1 also displays the top ten cited journals for relevant articles. The journal with the most citations was *Plos One* (1,462 citations), followed by *Nature* (1,026 citations), *Proceedings of the National Academy of Sciences* (PNAS) (902 citations), *Cell* (894 citations), and *Science* (699 citations). In all, 54 research areas were used to classify publications. Biochemistry and Molecular Biology was the field with the highest representation (67, 11.36%), followed by Microbiology (49, 8.30%), Pharmacology and Pharmacy (44, 7.46%), Gastroenterology and Hepatology (44, 7.46%), Immunology (36, 6.10%) and Multidisciplinary Sciences (36, 6.10%) (Table 2).

**TABLE 1:** Top 10 popular journals and cited journals.

Rank	Popular journals	Records (n)	2020 impact factor	2020 JCR partition	Cited journals	Citations (n)	2020 impact factor	2020 JCR partition
1	International journal of molecular sciences	20	5.924	Q1	Plos one	1,462	3.240	Q2
2	Frontiers in microbiology	15	5.640	Q1	Nature	1,026	49.962	Q1
3	Scientific reports	13	4.380	Q1	Proceedings of the National Academy of Sciences of the United States of America	902	11.205	Q1
4	Plos one	11	3.240	Q2	Cell	894	41.584	Q1
5	Frontiers in immunology	10	7.561	Q1	Science	699	47.728	Q1
6	Gastroenterology	8	22.682	Q1	Gastroenterology	619	22.682	Q1
7	Nutrients	8	5.719	Q1	Nucleic acids res	589	16.971	Q1
8	BMC genomics	7	3.969	Q2	Scientific reports	586	4.380	Q1
9	Applied microbiology and biotechnology	6	4.813	Q1	Gut	505	23.059	Q1
10	Probiotics and antimicrobial proteins	6	4.609	Q2	J bio chem	494	5.157	Q2

**TABLE 2:** Top ten well-represented research areas.

Rank	Research areas	Records (n)	% (of 590)
1	Biochemistry and molecular biology	67	11.36
2	Micobiology	49	8.30
3	Pharmacology and pharmacy	44	7.46
4	Gastroenterology and hepatology	44	7.46
5	Immunology	36	6.10
6	Multidisciplinary sciences	36	6.10
7	Genetics and heredity	34	5.76
8	Cell biology	28	4.75
9	Oncology	28	4.75
10	Medicine, research, and experimental	24	4.07

### 3.4 Analysis of authors

**FIGURE 5:**
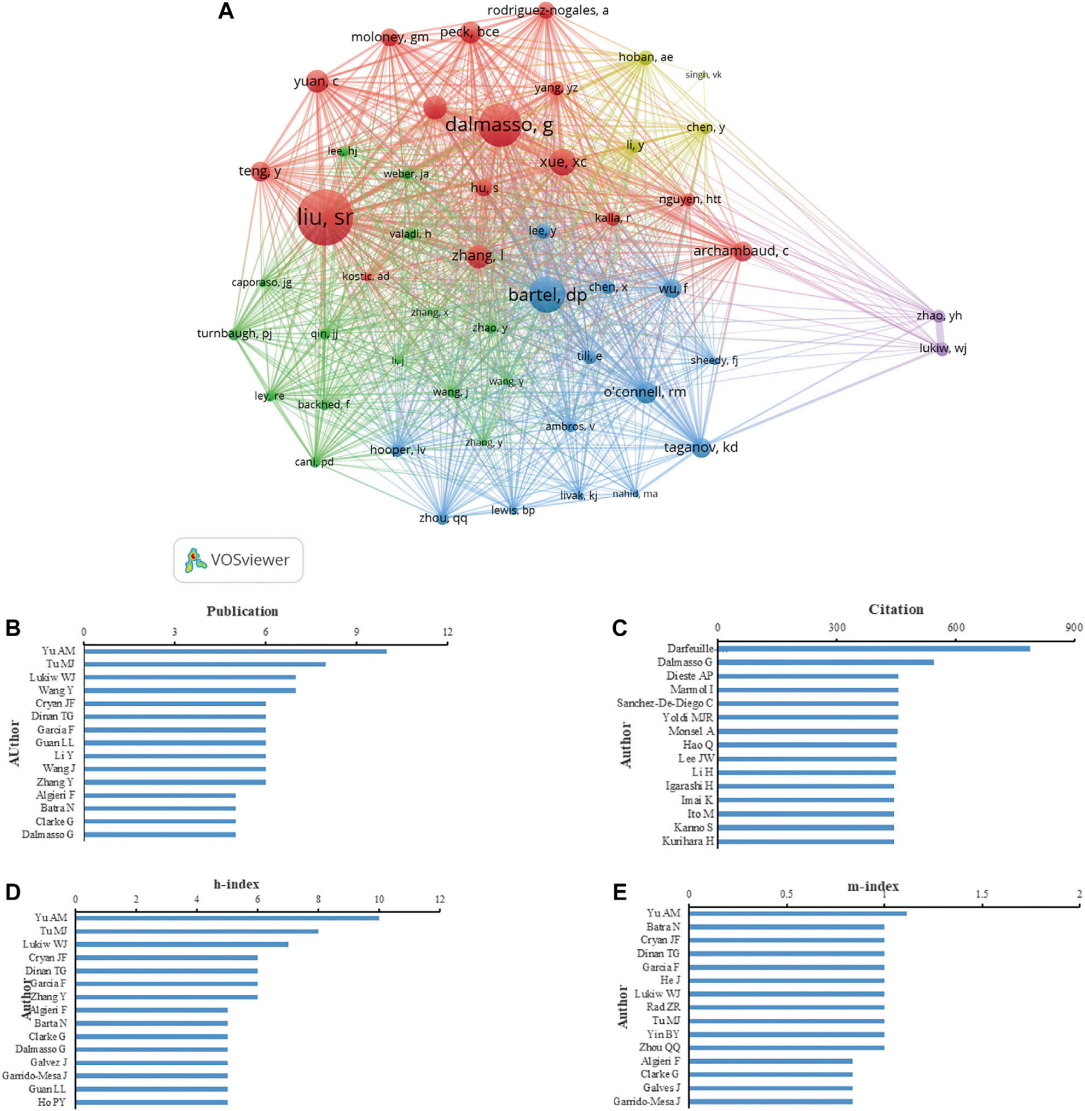
Analysis of authors focus on miRNA-microbiome interaction research. **(A)** VOSviewer’s network analysis of authors’ co-citation with more than 25 citations by VOSviewer. **(B)** Top 15 authors in the number of publications. **(C)** Top 15 authors in total citations. **(D)** Top 15 authors in h-index of publications. **(E)** Top 15 authors in m-index of publication.

According to the number of publications, Yu AM. was the most prolific author with 10 publications (1.69% of all publications), followed by Tu M. J. (8, 1.36%), Lukiw W. J. (7, 1.19%), Guan L. L. (6, 1.02%) and Dalmasso G. (6, 1.02%) (Figure 5B). According to authors’ citations analysis, Darfeuille M. A. ranked first (789 citations), followed by Dalmasso G. (546 citations), Cerrada E. (455 citations), Dieste A.P. (455 citations), Marmol I. (455 citations), Sanchez-De-Diego C. (455 citations), and Yoldi, M. J. R. (455 citations) (Figure 5C). Publications from Yu A.M. earned the highest h-index (10), followed by that from Tu M. J. (8), Lukiw W. J. (7), Cryan J. F. (6), Dinan T. G. (6), Garcia F. (6) and Zhang Y. (6) (Figure 5D). The m-index of publications from Yu AM (1.11) was also ranked first, followed by that from Batra N. (1.00), Cryan JF (1.00), Dinan T.G. (1.00), and Garcia F. (1.00) (Figure 5E). Moreover, 51 authors that were cited in more than 25 citations were analyzed. The top five authors with the highest total link strength were Liu S. R. (1,262), Dalmasso G. (934), Bartel D. P. (784), Xue X. C. (535), and Zhang L. (504) (Figure 5A).

### 3.5 Citation and co-citation analysis

**FIGURE 6:**
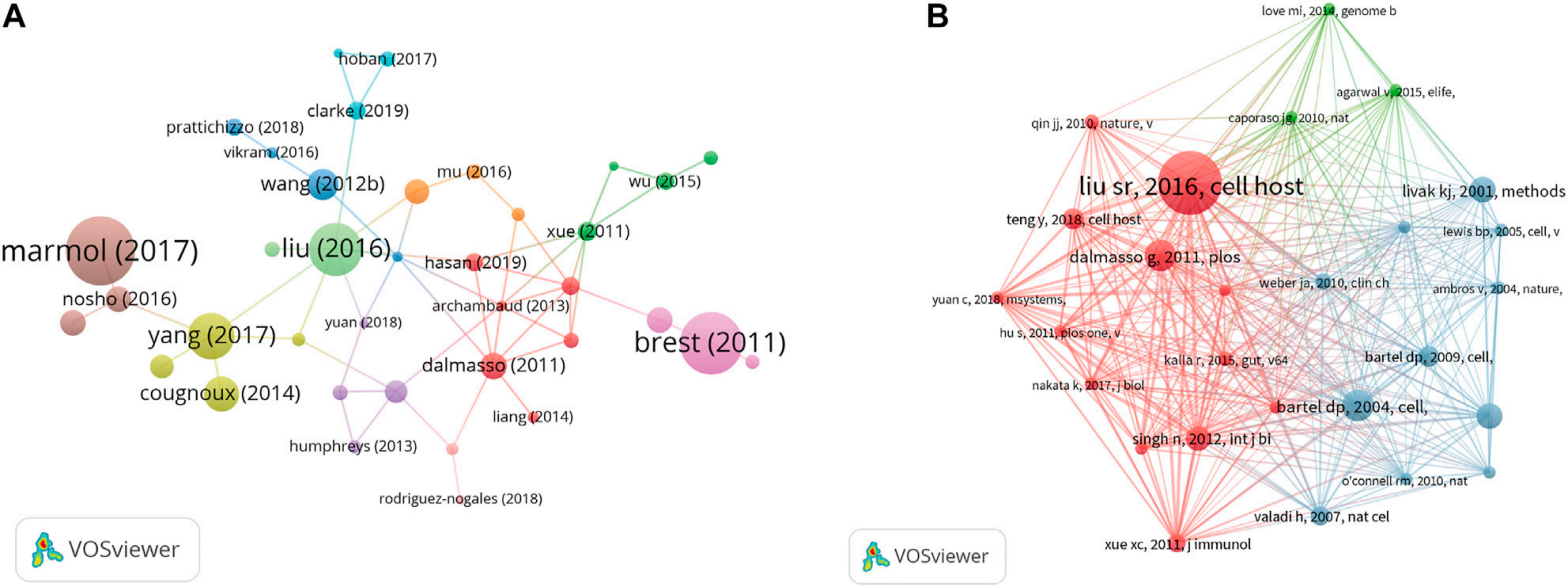
**(A)** VOSviewer’s network analysis of documents’ citation with more than 50 citations. **(B)** VOSviewer’s network analysis of references’ co-citation with more than 20 citations.

According to the analysis of citation, 70 documents contained more than 50 citations (Figure 6A). The top 10 documents with the most citations are shown in Table 3. There were 455 citations for “Colorectal Carcinoma: A General Overview and Future Perspectives in Colorectal Cancer” (Marmol et al., 2017), followed by “A synonymous variant in IRGM alters a binding site for miR-196 and causes deregulation of IRGM-dependent xenophagy in Crohn’s disease” (Brest et al., 2011), with 403 citations. The article with the third-highest amount of citations was “Human Mesenchymal Stem Cell Microvesicles for Treatment of *E. coli* Endotoxin-Induced Acute Lung Injury in Mice” (Zhu et al., 2014), with 403 citations too. In addition, we analyzed 27 references that were co-cited in excess of 20 times (Figure 6B). And the top three references with the largest number of citations were published by Liu S. R., (2016, *Cell Host and Microbe*; 106 citations), Bartel D. P., (2004, *Cell*; 53 citations), and Dalmasso G., (2011, *Plos One*; 53 citations).

**TABLE 3:** Top ten citation analysis of documents on miRNA-microbiome interaction research.

Rank	Title	First author/year	Source	Citations
1	Colorectal carcinoma: A general overview and future perspectives in colorectal cancer	Marmol L./2017	International journal of molecular sciences	455
2	A synonymous variant in IRGM alters a binding site for miR-196 and causes deregulation of IRGM-dependent xenophagy in Crohn’s disease	Brest, P./2011	Nature genetics	403
3	Human mesenchymal stem cell microvesicles for treatment of *E. coli* endotoxin-induced acute lung injury in mice	Zhu, Y. G./2014	Stem cells	403
4	The host shapes the gut microbiota *via* fecal microRNA	Liu, S. R./2016	Cell host and microbe	342
5	*F. nucleatum* increases proliferation of colorectal cancer cells and tumor development in mice by activating toll-like receptor 4 signaling to nuclear factor-kappa B, and upregulating expression of microRNA-21	Yang, Y. Z./2017	Gastroenterology	290
6	Bacterial genotoxin colibactin promotes colon tumour growth by inducing a senescence-associated secretory phenotype	Cougnoux, A./2014	Gut	213
7	Gut microbiota metabolism of anthocyanin promotes reverse cholesterol transport in mice *via* Repressing miRNA-10b	Wang, D. L./2012	Circulation research	192
8	Genome-wide antisense transcription drives mRNA processing in bacteria	Lasa, I./2011	Proceedings of the National Academy of Sciences of the United States of America	174
9	Mother’s milk: A purposeful contribution to the development of the infant microbiota and immunity	Le Doare, K./2018	Frontiers in immunology	171
10	New insights into oxidative stress and inflammation during diabetes mellitus-accelerated atherosclerosis	Yuan, T./2019	Redox biology	161

### 3.6 Co-occurrence and cluster analysis of keywords

**FIGURE 7:**
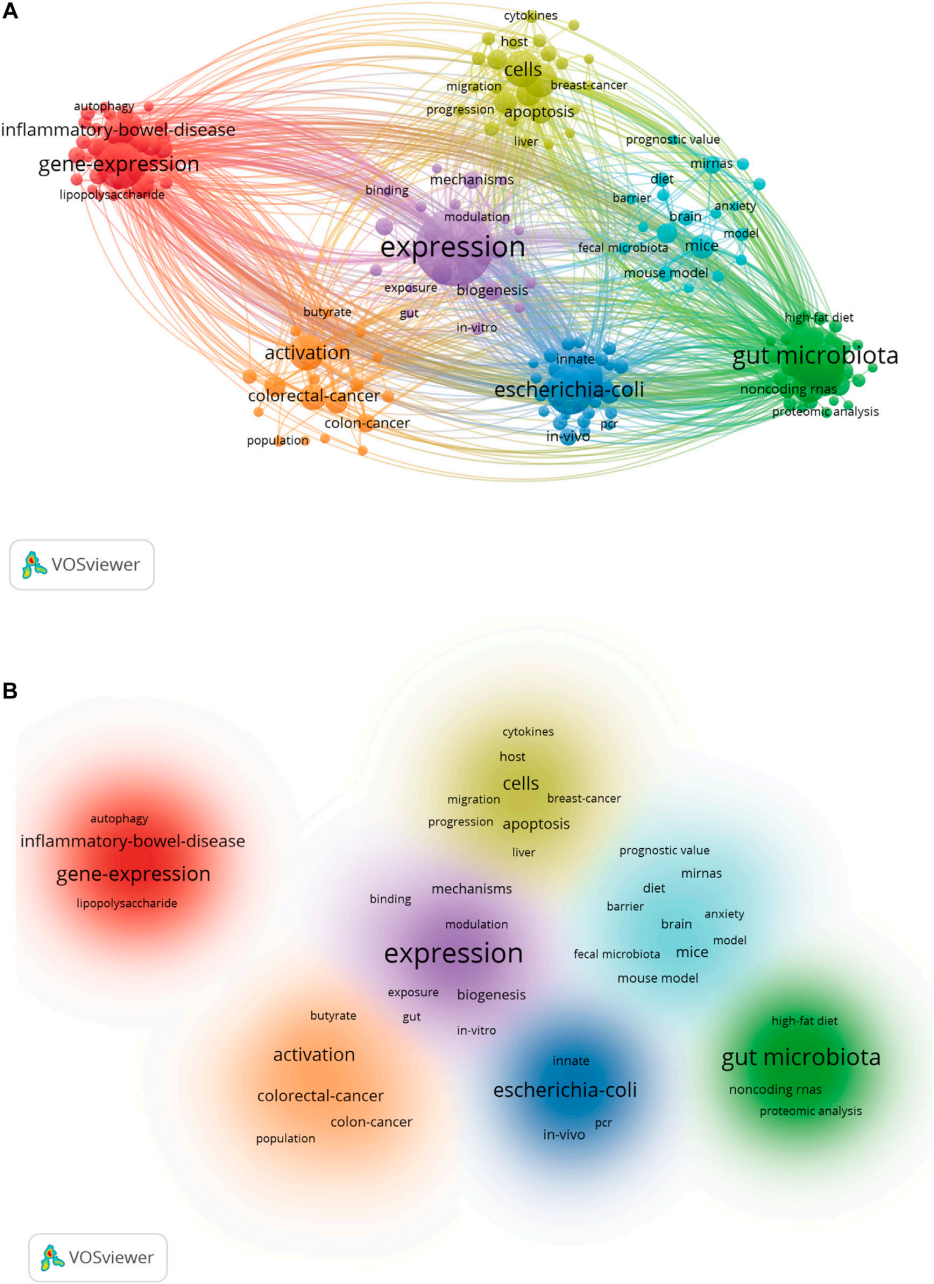
Keyword co-occurrence and cluster analysis. **(A)** VOSviewer’s network analysis of keywords. Distribution of keywords according to the mean frequency of appearance. **(B)** The clustering map based on occurrence analysis was divided into seven parts.

A total of 195 keywords that were discovered to have appeared more than five times were analyzed (Figure 7A). The five keywords with the highest frequency were expression (151 occurrences), gut microbiota (99), *E. coli* (68), gene expression (65), and microRNAs (61). Table 4 list the top twenty keywords with the highest frequency. Finally, according to the specific algorithm, they gathered into seven effective clusters corresponding to different colors. (Figure 7B).

**TABLE 4:** Top twenty occurrence analysis of keywords on miRNA-microbiome interaction research.

Rank	Keyword	Frequency	Total link strength	Rank	Keyword	Frequency	Total link strength
1	Expression	151	642	11	Identification	35	151
2	Gut microbiota	99	398	12	Inflammatory-bowel-disease	33	150
3	*E. coli*	68	264	13	Ulcerative-colitis	30	139
4	Gene expression	65	256	14	Colorectal cancer	26	128
5	MicroRNAs	61	276	15	Biomarkers	25	125
6	Cells	43	201	16	Nf-kappa-b	25	116
7	Activation	41	198	17	Proliferation	24	122
8	Inflammation	40	184	18	Epithelial-cells	22	104
9	Cancer	37	186	19	Mice	22	102
10	Disease	36	146	20	Association	21	106

## 4 Discussion

### 4.1 General trends

This study used bibliometric analysis and network visualization to conduct a comprehensive review of research progress on the interaction between miRNA and the microbiome, identifying hot topics within this field and predicting potential directions for future research.

590 articles and reviews spanning 2011–2021 were retrieved. The polynomial-fitting curve showed an overall upward trend for the number of annual publications; 79% of all identified contributions were published in the latter half of the study period. Furthermore, 142 studies were published in 2021, and the curve predicted that more than 200 studies will be published in 2023; this indicates strong growth in the annual publication output in the field.

A visual examination of the distribution of countries and institutions revealed that the United States contributed the largest number of citations and had the greatest number of co-authors, indicating that the United States is the epicenter of research on the interplay between miRNA and the microbiome. China had the greatest number of publications and the second-most citations, indicating that China has made a significant impact on the field. Moreover, China and the United States had strong cooperative relations. Shanghai Jiaotong University and the University of California Davis were the most productive institutions, and they focused on producing miRNA pharmaceuticals for cancer treatment by *in vivo* fermentation and identifying the mechanism of miRNA–microbiome interaction (Wang et al., 2015; Yang et al., 2017; Ho et al., 2018; Zhu et al., 2020). A paper published in the *International Journal of Molecular Sciences* had the highest number of citations, and this journal also had the highest number of records. The journals with the highest number of records and citations may be prioritized when researchers read and publish articles on miRNA–microbiome interactions, suggesting that the International Journal of Molecular Sciences played a key role in the field. The fields of biochemistry and molecular biology were the most represented regarding miRNA–microbiome research, and these fields are developing rapidly. This will likely enhance research related to miRNA–microbiome interactions.

### 4.2 Influential authors and studies

The h-index was used to assess the scientific research output and citation effect of researchers, while the m-index was intended to ease comparisons across researchers with varied academic careers. Yu A. M et al. (2019) had the largest number of publications and was the top-ranked author according to the h-index and m-index, indicating that he was a leader in terms of academic influence in the area of miRNA–microbiome research. Yu et al. (2020) have focused on developing novel approaches for the efficient production of non-coding RNAs using *in vivo* fermentation for targeted cancer treatment. They produced hsa-mir-27b, miR-27b-3p, and miR-328-3p in *E. coli* using recombinant RNA technology, providing a basis for future research on novel pharmaceuticals based on miRNA produced *via in vivo* fermentation. Liu et al. (2016) had the largest number of co-citations, indicating that he has played a pioneering role in miRNA–microbiome research. He identified that fecal miRNA from the host could enter the bacteria, affecting bacteria gene transcription and growth. Furthermore, Liu et al. (2019) revealed that fecal miRNA transplantation might aid in the restoration of gut microbiota and thus may be applied for clinical therapeutic. Darfeuille M. A. had the highest number of citations (789) and focused on susceptibility genes associated with autophagy in the etiology of Crohn’s disease and their intricate interaction with the gut microbiota (Brest et al., 2011; Lapaquette et al., 2012). These articles suggest that miRNAs and gut microbiota are likely to become physiological or pathological diagnostic markers for intestinal diseases in clinical practice.

We identified 70 documents with more than 50 citations. The records with the three highest numbers of citations focused on the effects of miRNA–microbiome interplay on different diseases. The authors of these papers observed that miRNA–microbiome interactions impacted the regulation of host gene expression involved in colorectal cancer, IBD, and acute lung injury through several mechanisms, including downregulation of autophagy-related proteins, suppression of inflammatory cytokine and chemokine secretion, and alteration of the abundance of microbiome metabolites. The reference with the most co-citations was published by Liu et al. (2016) in *Cell Host & Microbe*. The authors demonstrated that gut-derived miRNAs were involved in bidirectional interspecies gene regulation that contributed to the makeup of gut microbiota. In recent years, the references with the highest citation frequencies have focused on gene engineering (Yuan et al., 2018), targeted therapy (Rajesha and Frank, 2017), and tumors (Hu et al., 2015). The groundbreaking findings of the top authors and studies in the field of miRNA–microbiome interplay indicate that research has made several breakthroughs in the diagnosis and treatment of cancer by elucidating the mechanisms of miRNA–microbiome interactions.

### 4.3 Hotspots and frontiers

Keywords can be used to present and summarize the central argument and core content of the literature. This study used keyword co-occurrence analysis to explore the distribution and development of research hotspots in the field (Jie et al., 2021). The keywords that appeared more than 20 times, including “miRNA” and “gut microbiota,” are shown in Table 4. A clustering analysis was performed on the basis of keyword co-occurrence analysis to create a clustering map, which was divided into the following areas: physiological function (gene expression, autophagy, and apoptosis), diseases (cancer, IBD, et al.), and pharmacology (probiotic, *in vivo*, and *E. coli*).

#### 4.3.1 miRNA–microbiome interaction in regulating host gene expression

Studies have shown that miRNAs are implicated in microbiota-mediated control of host gene expression (Mody et al., 2021). Dalmasso and his team identified nine miRNAs in the ileum and colon of colonized mice that were expressed differently from those found in germ-free mice (Dalmasso et al., 2011). Experiments *In vitro* indicated that the downregulation of one of these miRNAs, mmu-miR-665, upregulated the expression of the target gene *Abcc3* during colonization, suggesting that miRNA may be implicated in gut microbiota-regulated changes in host gene expression (Dalmasso et al., 2011). Other studies have found that the gut microbiota can modulate host immune responses *via* miRNAs. For example, bacteria were shown to inhibit the production of miR-10a in dendritic cells through TLR–TLR ligand interactions mediated by a MyD88-dependent mechanism (Xue et al., 2011). miR-155, which is involved in TLR activation *via* bacteria-derived lipopolysaccharides, was shown to activate tumor necrosis factor (TNF)-α and interleukin (IL)-6 and regulate suppressor of cytokine signaling 1 (SOCS1) on dendritic cells, thus playing a role in adaptive immune responses (Esmerina et al., 2007; Naqvi et al., 2014). In addition, bacteria can secrete extracellular vesicles that carry extracellular miRNAs; these vesicles participate in intercellular communication to reach remote target cells (Yu S. R et al., 2019; Badi et al., 2020; Stanton, 2021). For example, the probiotic *E. coli* Nissle 1917 strain produces outer-membrane vesicles that control the expression of the ZO-1 and ZO-2 tight junction proteins, enhancing intestinal immune regulation and barrier function (Veltman et al., 2012; Sabharwal et al., 2016).

**FIGURE 8:**
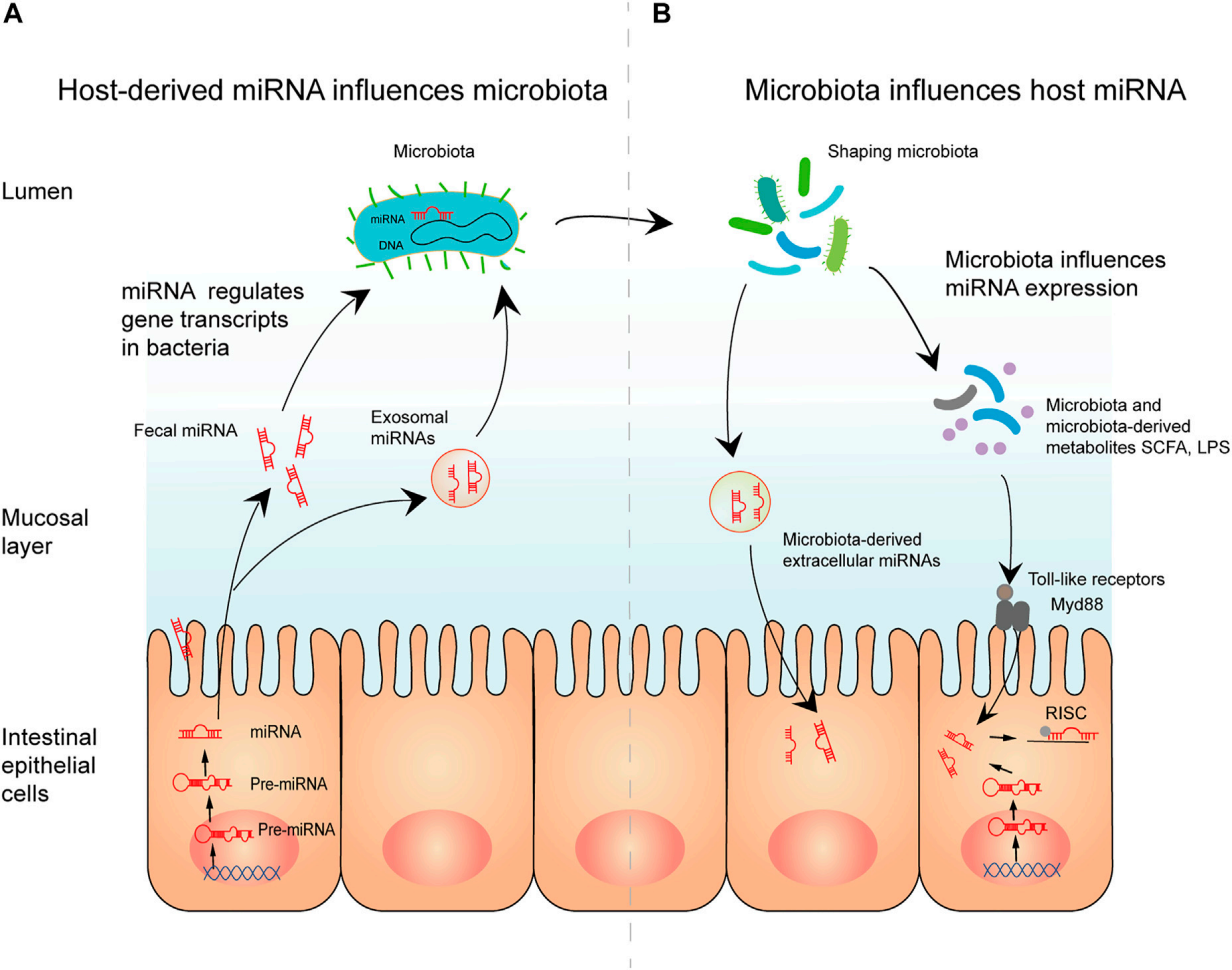
The mechanisms of interplay between miRNA and the microbiome. **(A)** Host-derived miRNA influences microbiota. Mature miRNAs from intestinal epithelial cells can enter microbiota in the form of fecal miRNA or exosomal miRNA. By regulating gene transcription in microbiota, miRNA affects microbial growth, thereby shaping the microbiota. **(B)** Microbiota influences host miRNA. Microbiota and microbiota-derived metabolites regulate miRNA expression, possibly through toll-like receptor/Myd88-dependent pathways. Moreover, microbiota-derived extracellular vesicle can modulate gut barrier function and the immune response directly.

Conversely, host miRNA may also regulate gut microbiota colonization and gene expression. In 2016, Liu et al. (2016) reported that fecal miRNAs may influence the composition of the gut microbiota. First, they used 16sRNA sequencing to identify the fecal bacteria of wild-type mice and Dicer1ΔIEC mice, which revealed substantial variations between the two groups in the species and abundance of the fecal bacteria. Subsequent *in vitro* studies found that miR-515-5p and miR-1226-5p promoted the growth of *F. nucleatum* and *E. coli*, respectively, demonstrating that miRNAs directly affect bacterial growth. Further investigation revealed that fecal miRNA could affect intestinal microorganisms. The fecal miRNA of wild-type mice was extracted and transplanted into intestinal epithelial cell (IEC) miRNA-deficient (Dicer1ΔIEC) mice, which exhibit dysfunctional gut microbiota (Moloney et al., 2018; Lei et al., 2019; Bi et al., 2020; Cheng et al., 2021). Interestingly, the gut microbiome of Dicer1ΔIEC mice was restored after transplantation, with species composition and abundance similar to those of the microbiome of wild-type mice (Monaghan et al., 2021; Zhang et al., 2021). Therefore, fecal miRNA transplantation appears to have therapeutic potential. In addition, recent findings have demonstrated that host-secreted miRNAs could regulate gene transcripts in bacteria (such as *F. nucleatum* and *E. coli*) and affect their growth, supporting the above research results. Figure 8 showed the mechanisms of interplay between miRNA and the microbiome (Liu et al., 2016; Yun et al., 2018; Santos et al., 2020). In brief, these studies demonstrate that bidirectional modulation between the gut microbiota and miRNAs regulates host gene expression through various pathophysiologic pathways and microbiome-derived metabolites (Table 5).

**TABLE 5:** MicroRNA-microbiota interaction in host pathophysiology.

Microbiota/miRNAs	Target	Effect	Function	Reference
*E. coli* Nissle 1917	miR-155, miR-223, miR-150, miR-203, miR-483-3p, miR-595	Downregulation	Anti-inflammation	Rodriguez-Nogales et al. (2018a)
	miR-143, miR-375	Upregulation	Barrier-enforcing	Veltman et al. (2012)
Enteropathogenic *E. coli*	miR-146a, miR-155, miR-21	Upregulation	Pro-inflammation	Sabharwal et al. (2016)
*L. fermentum*	miR-150, miR-155, miR-223	Downregulation	Anti-inflammation barrier-enforcing	Rodriguez-Nogales et al. (2017)
	miR-143	Upregulation		
*L. salivarius*	miR-155, miR-223	Downregulation	Anti-inflammation barrier-enforcing	Rodriguez-Nogales et al. (2017)
*F. nucleatum*	miR-21	Upregulation	Pro-inflammation	Yang et al. (2017)
	miRNA-18a	Downregulation		
	miRNA-4802		Promote CRC cells proliferation	
*Lactobacillus*	miR-155, miR-135b	Downregulation	Anti-inflammation	Heydari et al. (2019)
*Bifidobacterium*		Upregulation		
	miR-26b, miR-18a			
has-miR-515-5p	*F. nucleatum*	Promoting bacterial growth	Regulate specific bacterial gene expression	Liu et al. (2016)
has-miR-1226-5p	*E. coli*	Promoting bacterial growth	Regulate specific bacterial gene expression	Liu et al. (2016)

#### 4.3.2 Interplay between miRNA and the microbiome in diseases

##### 4.3.2.1 Colorectal cancer

Colorectal cancer (CRC) ranks as the third-leading cause of cancer-related death worldwide and represents approximately 10% of all tumors every year (Marmol et al., 2017). Multiple studies have established that an imbalance in the gut microbiota contributes to tumor cells invasion and metastasis (Tania et al., 2020; Jaeho and Heung, 2021) and that miRNAs can intervene in the genesis of CRC by mediating the microbiome (He et al., 2017; Yuan and Subramanian, 2019; Peruhova et al., 2020). Patients with early-stage CRC showed a significant increase in *F. nucleatum*, *Bacteroides fragilis*, *E. coli*, and *Enterococcus faecalis*, accompanied by increased levels of inflammatory factors and macrophage inflammatory protein 3A (MIP3A) in the serum (Aleksandar et al., 2013; Yuan et al., 2018). Additionally, miR-20a, miR-21, miR-96, miR-182, miR-183, and miR-7974 were dramatically overexpressed in cancerous tissues, suggesting that cancer-specific miRNAs are modulated in the early stages of the disease (Yuan et al., 2018). Recently, *F. nucleatum* was found to upregulate the expression of miR-21, initiating TLR4–MyD88 activation and augmenting CRC cell proliferation (Yang et al., 2017). In addition, the gut microbiota plays a critical role in the regulation of cancer metastasis through the IL11/circular RNA/miRNA/SOX9 axis (Zhu et al., 2020). Thus, miRNA–microbiome interaction plays a critical role in CRC development, and *F. nucleatum* miR-21 is suggested to be a promising diagnostic and prognostic biomarker for CRC.

Further research found that both endogenous and exogenous miR-139-5p may limit the proliferation of *F. nucleatum*-related CRC by provoking tumor-specific proteins, such as c-Myc and cyclin D1 (Zhao et al., 2020), demonstrating the potential therapeutic value of endogenous or exogenous miRNA in CRC. Furthermore, diet and metabolism, which affect the gut microbiota composition, also impact the development of CRC disease (Rajoka et al., 2018; Chen et al., 2021). Butyrate, a bacterial-derived metabolite, can reduce the expression levels of miR-92a, consequently inhibiting the proliferation of colon cancer cells and stimulating apoptosis (Hu et al., 2015; Ali et al., 2021). Together, these results indicate new diagnostic and prognostic biomarkers, and therapeutic targets for CRC patients.

##### 4.3.2.2 Inflammatory bowel disease

IBD is a term used to describe chronic inflammatory disorders of the gastrointestinal tract, including Crohn’s disease (CD) and ulcerative colitis (UC). Recently, a growing body of evidence has suggested that inflammation is connected to the overexpression of miRNA-21, which is positively correlated with histologically assessed disease severity (Dai et al., 2015; Mallet et al., 2021; Mikkel et al., 2021; Nivedita et al., 2022; Xiaoran et al., 2022). Moreover, Johnston et al. used 16s rRNA sequencing analysis to confirm altered microbial composition in miR-21^−/−^ colitis model mice, including an increase in the number of *Firmicutes* and a decrease in the number of the phylum *Bacteroidetes* (Johnston et al., 2018). This suggests that miR-21 affects the etiology of intestinal inflammation by altering intestinal microbiota composition (Joana et al., 2018).

In patients with IBD, the expression of miR-10a is negatively regulated by microbiome *via* a MyD88-dependent pathway, promoting intestinal homeostasis (Xue et al., 2011). Other inflammatory pathways have also been reported in the past decade. IBD-associated adherent invasive *E. coli* (AIEC) has been linked to IBD pathology as a cause or contributing factor (Carolina et al., 2017). AIEC-induced IBD upregulated the levels of miR-30C and miR-130A by activating the NF-kb pathway, reducing the expression of proteins related to autophagy (Larabi et al., 2020). Moreover, the miRNA let-7b was found to effectively reduce proinflammatory cytokines in AIEC-infected mice by regulating TLR-4 expression, suggesting that let-7b is a prospective therapeutic target for IBD, especially AIEC-induced IBD (Guo et al., 2018). Recently, exosomal miR-181a from mesenchymal stem cells was demonstrated to alleviate inflammation and promote intestinal barrier function by decreasing TNF-α, IL-6, IL-1β, IL-17, and IL-18 levels; increasing claudin-1 and ZO-1 levels; and affecting the gut microbiota (Gu et al., 2021). This indicates that miR-181a may exhibit the potential to treat dysfunction of the intestinal mucosal barrier.

In addition, the probiotics *Lactobacillus fermentum*, *Lactobacillus salivarius*, and *E. coli* Nissle 1917 were shown to modulate miRNAs (miR-143, miR-150, miR-155, miR-223, and miR-375) in a colitis mouse model and reverse the disruption of the intestinal immune barrier (Rodriguez-Nogales et al., 2017; Rodriguez-Nogales et al., 2018a; Rodriguez-Nogales et al., 2018b). Thus, miRNA–microbiome interactions hold potential for effective diagnostic, preventive, and therapeutic methods in IBD.

##### 4.3.2.3 Neurological disorders

Gut microbes are key signaling components in the bidirectional communication between the gut and the brain (Emeran et al., 2015). Recent research described the critical roles of miRNAs in regulating gut–brain axis functions (Gerard et al., 2019; Singh et al., 2021). Furthermore, an imbalance in miRNA–microbiome function is associated with the appearance and evolution of neurological disorders, such as stress and anxiety, Parkinson’s disease (PD), Alzheimer’s disease (AD), and multiple sclerosis (MS) (Zhao and Lukiw, 2018; Moloney et al., 2019; Seo and Anderson, 2019).

Using in silico target screening, Hewel et al. identified over 300 commonly dysregulated miRNAs involved in bacterial pathways connected to AD and PD, including hsa-miR-1183, hsa-miR-3916, hsa-miR-1538, hsa-miR-3180-5p, hsa-miR-1248, hsa-miR-4767, hsa-miR-1301-3p, hsa-miR-378c, hsa-miR-671-5p, and hsa-miR-939-5p (Hewel et al., 2019). Alternatively, miRNA–microbiome interactions may impact different parts of the brain related to behavior and cognition. In germ-free mice, 134 dysregulated miRNAs were detected in the amygdala and the prefrontal cortex; dysfunction in these brain regions can lead to fear- and anxiety-like disorders. However, the increased expression of miR-294-5p in the hippocampus of germ-free mice normalized upon recolonization (Hoban et al., 2017). This miRNA targets and regulates the kynurenine metabolic pathway, demonstrating the crucial role of miRNA–microbiome interaction in neurological disorders and presenting an opportunity for the development of potential treatment (Moloney et al., 2017).

A recent study reported that oral administration of *Lactobacillus* could reduce the expression of miR-155 and increase the expression of miR-25 by reversing the effect of cuprizone and alleviating demyelinating symptoms in MS (Digehsara et al., 2021). Together, these studies suggest that probiotics and miRNAs have the potential to improve the prevention, treatment, and prognosis of neurological diseases.

#### 4.3.3 Pharmacology of miRNA–microbiome interplay

Because of the role of miRNA–microbiome interplay in modulating physiological and pathological conditions, therapeutic strategies such as dietary change, probiotic supplementation, fecal transplantation, and miRNA drug administration are promising (Masterson et al., 2020; Mallet et al., 2021; Mao et al., 2021; Monaghan et al., 2021). In pharmacology, probiotics (such as *Bifidobacterium* and *Lactobacillus*) have been widely used to treat diseases such as cancer, neurological disorders, and IBD (Ahmed et al., 2019; Hasan and Yang, 2019; Heydari et al., 2019). However, miRNA drugs have not been developed for clinical use because they are endogenous, have multiple targets, and show poor biological stability (Aditya et al., 2017; Schmidt, 2017; Moloney et al., 2017). The strategies currently used to manipulate miRNAs typically substitute or upregulate the expression of targeted miRNA or downregulate the expression of mature miRNAs to inhibit them. Studies have revealed that *in vitro* technologies, such as miRNA mimics, agomirs, precursors containing stem-loops, and viral vector constructs, could upregulate gene expression (Bianca et al., 2015; Sarah and Gyorgy, 2020), whereas anti-miRNA oligonucleotides, antagomirs, and miRNA sponges could downregulate gene expression (Kesley et al., 2015; Johnston et al., 2018). Because of the problems that arise when miRNA drugs are transported from the *in vitro* environment to target cells (degradation, off-target effects, and toxicity), researchers have begun to produce miRNA drugs using *in vivo* fermentation.

Yu A. M. et al. (2019) produced a variety of RNA vectors that can be expressed as endogenous molecules. These molecules continuously accumulate and express *in vivo* and then form recombinant RNA molecules in bacteria to regulate the expression of target genes (Li et al., 2014; Li et al., 2015). This fermentation technology is a consistent, efficient, and cost-effective method to produce various biological RNA reagents (Zhang et al., 2018; Yu et al., 2020). In addition, they used fermentation technology to produce bioengineered RNA agents. In human tumor LS-180 cells, BERA/mir-27b-3p was processed into mature mir-27b-3p, which made the tumor cells sensitive to chemotherapeutic drugs (Li et al., 2019). In addition, Sun et al. (2017) developed a bacteriophage PP7 virus-like particle (VLP)-based delivery system without infectious, replicative, or cytotoxic effects. They found that recombinant PP7 VLPs carrying a cell-penetrating peptide (CPP) and miRNA were more efficiently expressed in *E. coli* than other miRNA delivery methods; therefore, they could be used as an effective and stable delivery vector of miRNA. Overall, *in vivo* fermentation technology has the potential to be used for genomic diagnosis and treatment and could be validated for clinical use in the future.

### 4.4 Limitations

Although we followed bibliometric principles and comprehensive analysis strategies, our research has several limitations. First, our study is restricted to the WoSCC database, resulting in the loss of any studies not included in the WoSCC. Future studies utilizing more databases (such as Scopus and Pubmed) with wider coverage are strongly encouraged. Second, although the study period of 2011–2021 reflects the major research breakthroughs and hotspots, some original papers that made groundbreaking contributions to this field, along with newly published papers, may have been overlooked. Third, all information was extracted by the R package bibliometrix and VOSviewer, which may be biased by the results of other bibliometric tools.

## 5 Conclusion

This bibliometric study revealed that the number of publications in the field of miRNA–microbiome research in the past decade has increased continuously and rapidly. China was a major producer of studies, and the USA had a great influence in this field. The interaction between the microbiome and miRNA regulates host gene expression and is implicated in the pathogenesis of many groups of diseases, such as cancer (colorectal cancer, et al.), IBD (CD and UC), and neurological disorders (anxiety, PD, AD, et al.), and this interaction has thus become a research hotspot. The literature suggests that miR-21, miR-155, miR-146a, *E. coli*, *Bifidobacterium*, *F. nucleatum*, and butyric acid have the potential to improve disease diagnosis, treatment, and prognosis. Our results indicate that future research may explore miRNA drugs produced by *in vivo* fermentation, a technology that can greatly increase the stability and reduce the degradation of miRNA.

## Data Availability

The original contributions presented in the study are included in the article/supplementary materials. The study data supporting the conclusions of this article will be shared openly, without undue reservation.

## References

[B1] Aditya G. Sheema K. Bilal B. H. Stephen W. B. Murali M. Y. Subhash C. C. (2017). miRNA nanotherapeutics for cancer. Drug Discov. Today 22 (2), 424424–432432. 10.1016/j.drudis.2016.10.014 PMC5309208 27815139

[B2] Ahmed B. Rasul A. Tareen K. Z. Akash M. S. H. Muhammad S. A. Irfan M. (2019). Contemporary evidence on the dynamic role of probiotics in liver diseases. Pak. J. Pharm. Sci. 32 (6), 2759–2764. 31969314

[B3] Aleksandar D. K. Eunyoung C. Lauren R. Jonathan N. G. Carey A. G. Monia M. (2013). Fusobacterium nucleatum potentiates intestinal tumorigenesis and modulates the. Cell Host Microbe 14 (2), 207207–215215. 10.1016/j.chom.2013.07.007 PMC3772512 23954159

[B4] Ali S. R. Orang A. Marri S. McKinnon R. A. Meech R. Michael M. Z. (2021). Integrative transcriptomic network analysis of butyrate treated colorectal cancer cells. Cancers 13 (4), 636. 10.3390/cancers13040636 33562636 PMC7914650

[B5] Badi S. A. Bruno S. P. Moshiri A. Tarashi S. Siadat S. D. Masotti A. (2020). Small RNAs in outer membrane vesicles and their function in host-microbe interactions. Front. Microbiol. 11, 1209. 10.3389/fmicb.2020.01209 32670219 PMC7327240

[B6] Bi K. F. Zhang X. J. Chen W. B. Diao H. Y. (2020). MicroRNAs regulate intestinal immunity and gut microbiota for gastrointestinal health: A comprehensive review. Genes 11 (9), E1075. 10.3390/genes11091075 32932716 PMC7564790

[B7] Bianca C. B. Jenny Y. O. Ruby C. L. Julie R. M. (2015). miRNA therapeutics: a new class of drugs with potential therapeutic applications. Future Med. Chem. 7 (13), 17711771–17921792. 10.4155/fmc.15.107 26399457

[B8] Brest P. Lapaquette P. Souidi M. Lebrigand K. Cesaro A. Vouret-Craviari V. (2011). A synonymous variant in IRGM alters a binding site for miR-196 and causes deregulation of IRGM-dependent xenophagy in Crohn's disease. Nat. Genet. 43 (3), 242–245. 10.1038/ng.762 21278745

[B9] Cao Y. Y. Wang Z. H. Yan Y. Q. Ji L. H. He J. Xuan B. Q. (2021). Enterotoxigenic Bacteroides fragilis promotes intestinal inflammation and malignancy by inhibiting exosome-packaged miR-149-3p. Gastroenterology 161(5), 1552-1566. 10.1053/j.gastro.2021.08.003 34371001

[B10] Carolina P. Caroline C. Zhilu X. Joana T. Gwladys S. Robert H. (2017). - Adherent-invasive *Escherichia coli* in inflammatory bowel disease. Gut 67 (3), 574574–587587. 10.1136/gutjnl-2017-314903

[B11] Casado-Bedmar M. Viennois E. (2021). MicroRNA and gut microbiota: Tiny but mighty-novel insights into their cross-talk in inflammatory bowel disease pathogenesis and therapeutics. J. Crohns Colitis 14, 992–1005. 10.1093/ecco-jcc/jjab223 PMC9282881 34918052

[B12] Chen Y. S. Li J. Menon R. Jayaraman A. Lee K. Huang Y. (2021). Dietary spinach reshapes the gut microbiome in an apc-mutant genetic background: mechanistic insights from integrated multi-omics. Gut Microbes 13 (1), 1972756. 10.1080/19490976.2021.1972756 34494932 PMC8437542

[B13] Cheng L. Q. Kazmierczak D. Norenhag J. Hamsten M. Fransson E. Schuppe-Koistinen I. (2021). A MicroRNA gene panel predicts the vaginal microbiota composition. Msystems 6 (3), e00175. 10.1128/mSystems.00175-21 33947805 PMC8269211

[B14] Dai X. Chen X. Chen Q. Shi L. Liang H. W. Zhou Z. (2015). MicroRNA-193a-3p reduces intestinal inflammation in response to microbiota via down-regulation of colonic PepT1. J. Biol. Chem. 290 (26), 16099–16115. 10.1074/jbc.M115.659318 25931122 PMC4481212

[B15] Dalmasso G. Hang T. T. N. Yan Y. T. Laroui H. Charania M. A. Ayyadurai S. (2011). Microbiota modulate host gene expression via MicroRNAs. Plos One 6 (4), e19293. 10.1371/journal.pone.0019293 21559394 PMC3084815

[B16] David P. B. (2004). MicroRNAs: genomics, biogenesis, mechanism, and function. Cell 116 (2), 281281–297297. (Print)). 10.1016/s0092-8674(04)00045-5 14744438

[B17] Deepansh M. Vedika V. Vibha, R. (2021). Modulating host gene expression via gut microbiome-microRNA interplay to treat. Crit. Rev. Microbiol. 47 (5), 596596–611611. 10.1080/1040841X.2021.1907739 34407384

[B18] Digehsara S. G. Name N. Esfandiari B. Karim E. Taheri S. Tajabadi-Ebrahimi M. (2021). Effects of Lactobacillus casei strain T2 (IBRC-M10783) on the modulation of Th17/treg and evaluation of miR-155, miR-25, and IDO-1 expression in a cuprizone-induced C57BL/6 mouse model of demyelination. Inflammation 44 (1), 334–343. 10.1007/s10753-020-01339-1 32914363

[B19] Emeran M. Kirsten T. Gupta A. (2015). Gut/brain axis and the microbiota. J. Clin. Invest. 125 (3), 926926–938938. 10.1172/jci76304 PMC4362231 25689247

[B20] Ernesto R.-V. Shirley S.-R. Rafael I.-C. Camilo R. (2019). Current concepts on bibliometrics: a brief review about impact factor. Ir. J. Med. Sci. 188 (3), 939939–951951. 10.1007/s11845-018-1936-5 30511320

[B21] Esmerina T. Jean-Jacques M. Amelia C. Stefan C. Calin Dan D. Brett A. (2007). Modulation of miR-155 and miR-125b levels following lipopolysaccharide/TNF-alpha. J. Immunol. 179, 5082. 10.4049/jimmunol.179.8.5082 17911593

[B22] Feng Q. Q. Chen W. D. Wang Y. D. (2018). Gut microbiota: An integral moderator in health and disease. Front. Microbiol. 9, 151. 10.3389/fmicb.2018.00151 29515527 PMC5826318

[B23] Gerard M. Timothy G. D. Gerard C. John F. C. (2019). Microbial regulation of microRNA expression in the brain-gut axis. Curr. Opin. Pharmacol. 48, 120120–126126. 10.1016/j.coph.2019.08.005 31590111

[B24] Gu L. Ren F. Fang X. R. Yuan L. W. Liu G. L. Wang S. L. (2021). Exosomal MicroRNA-181a derived from mesenchymal stem cells improves gut microbiota composition, barrier function, and inflammatory status in an experimental colitis model. Front. Med. 8, 660614. 10.3389/fmed.2021.660614 PMC8264068 34249964

[B25] Guo Z. Cai X. C. Guo X. Xu Y. H. Gong J. F. Li Y. (2018). Let-7b ameliorates Crohn's disease-associated adherent-invasive E coli induced intestinal inflammation via modulating Toll-Like Receptor 4 expression in intestinal epithelial cells. Biochem. Pharmacol. 156, 196–203. 10.1016/j.bcp.2018.08.029 30142321

[B26] Gupta I. Sehgal R. Kanwar R. K. Punj V. Kanwar J. R. (2015). Nanocapsules loaded with iron-saturated bovine lactoferrin have antimicrobial therapeutic potential and maintain calcium, zinc and iron metabolism. Nanomedicine 10 (8), 1289–1314. 10.2217/nnm.14.209 25442715

[B27] Hasan N. Yang H. Y. (2019). Factors affecting the composition of the gut microbiota, and its modulation. Peerj 7, e7502. 10.7717/peerj.7502 31440436 PMC6699480

[B28] He C. Yu T. M. Shi Y. Ma C. Y. Yang W. J. Fang L. L. (2017). MicroRNA 301A promotes intestinal inflammation and colitis-associated cancer development by inhibiting BTG1. Gastroenterology 152 (6), 1434–1448. 10.1053/j.gastro.2017.01.049 28193514

[B29] Hewel C. Kaiser J. Wierczeiko A. Jan L. K. Reinhardt C. Endres K. (2019). Common miRNA patterns of alzheimer's disease and Parkinson's disease and their putative impact on commensal gut microbiota. Front. Neurosci. 13, 113. 10.3389/fnins.2019.00113 30890906 PMC6411762

[B30] Heydari Z. Rahaie M. Alizadeh A. M. Agah S. Khalighfard S. Bahmani S. (2019). Effects of Lactobacillus acidophilus and Bifidobacterium bifidum probiotics on the expression of MicroRNAs 135b, 26b, 18a and 155, and their involving genes in mice colon cancer. Probiotics Antimicrob. Proteins 11 (4), 1155–1162. 10.1007/s12602-018-9478-8 30311185

[B31] Hirsch J. E. (2005). An index to quantify an individual's scientific research output. Proc. Natl. Acad. Sci. U. S. A. 102 (46), 16569–16572. 10.1073/pnas.0507655102 16275915 PMC1283832

[B32] Ho P. Y. Duan Z. J. Batra N. Jilek J. L. Tu M. J. Qiu J. X. (2018). Bioengineered noncoding RNAs selectively change cellular miRNome profiles for cancer therapy. J. Pharmacol. Exp. Ther. 365 (3), 494–506. 10.1124/jpet.118.247775 29602831 PMC5931433

[B33] Hoban A. E. Stilling R. M. Moloney G. M. Moloney R. D. Shanahan F. Dinan T. G. (2017). Microbial regulation of microRNA expression in the amygdala and prefrontal cortex. Microbiome 5, 102. 10.1186/s40168-017-0321-3 28838324 PMC5571609

[B34] Horne R. St Pierre J. Odeh S. Surette M. Foster J. A. (2019). Microbe and host interaction in gastrointestinal homeostasis. Psychopharmacology 236 (5), 1623–1640. 10.1007/s00213-019-05218-y 30900006 PMC6599184

[B35] Horvitz H. R. Sulston J. E. (1980). Isolation and genetic characterization of cell-lineage mutants of the nematode. - Genet. 96 (2), 435435–454454. 10.1093/genetics/96.2.435 PMC1214309 7262539

[B36] Hu S. E. Liu L. Chang E. B. Wang J. Y. Raufman J. P. (2015). Butyrate inhibits pro-proliferative miR-92a by diminishing c-Myc-induced miR-17-92a cluster transcription in human colon cancer cells. Mol. Cancer 14, 180. 10.1186/s12943-015-0450-x 26463716 PMC4604099

[B37] Jaeho K. Heung K. L. (2021). The role of gut microbiota in modulating tumor growth and anticancer agent. Mol. Cells 44 (5), 356356–362362. 10.14348/molcells.2021.0032 PMC8175145 33972463

[B38] James M. K. Ara W. D. Jeramy K. N. (2011). Gut microbiome-host interactions in health and disease. Genome Med. 3 (3), 14. 10.1186/gm228 21392406 PMC3092099

[B39] Jiayi D. Jesse W. T. Lu L. F. (2019). miRNA-microbiota interaction in gut homeostasis and colorectal cancer. Trends Cancer 5 (11), 666666–669669. 10.1016/j.trecan.2019.08.003 PMC8480531 31735285

[B40] Jie Z. Luxia S. Liyan X. Yixuan F. Tong W. Wende T. (2021). Knowledge domain and emerging trends in ferroptosis research: A bibliometric and. Front. Oncol. 11, 686726. 10.3389/fonc.2021.686726 34150654 PMC8209495

[B41] Joana F. L. Laura C. Ceu F. Carla O. Nuno F. A. (2018). - anti-miRNA oligonucleotides: A comprehensive guide for design. RNA Biol. 15 (3), 338338–352352. 10.1080/15476286.2018.1445959 PMC5927725 29570036

[B42] Johnston D. G. W. Williams M. A. Thaiss C. A. Cabrera-Rubio R. Raverdeau M. McEntee C. (2018). Loss of MicroRNA-21 influences the gut microbiota, causing reduced susceptibility in a murine model of colitis. J. Crohns Colitis 12 (7), 835–848. 10.1093/ecco-jcc/jjy038 29608690

[B43] Kathryn P. James V. (2012). Human microbiome in health and disease. Annu. Rev. Pathol. 7, 9999–122122. 10.1146/annurev-pathol-011811-132421 21910623

[B44] Kesley B. Chirostopher N. Craig L D. (2015). MiRNA inhibition in tissue engineering and regenerative medicine. Adv. Drug Deliv. Rev. 88, 123123–137137. 10.1016/j.addr.2014.12.006 PMC4485980 25553957

[B45] Lapaquette P. Brest P. Hofman P. Darfeuille-Michaud A. (2012). Etiology of Crohn's disease: many roads lead to autophagy. J. Mol. Med. 90 (9), 987–996. 10.1007/s00109-012-0934-8 22797958

[B46] Larabi A. Dalmasso G. Delmas J. Barnich N. Nguyen H. T. T. (2020). Exosomes transfer miRNAs from cell-to-cell to inhibit autophagy during infection with Crohn's disease-associated adherent-invasiveE. coli. Gut Microbes 11 (6), 1677–1694. 10.1080/19490976.2020.1771985 32583714 PMC7524154

[B47] Lei L. Yang Y. Wu S. Ma X. Mao M. Hu T. (2019). Mechanisms by which small RNAs affect bacterial activity. J. Dent. Res. 98 (12), 1315–1323. 10.1177/0022034519876898 31547763

[B48] Li M. M. Addepalli B. Tu M. J. Chen Q. X. Wang W. P. Limbach P. A. (2015). Chimeric MicroRNA-1291 biosynthesized efficiently in *Escherichia coli* is effective to reduce target gene expression in human carcinoma cells and improve chemosensitivity. Drug Metab. Dispos. 43 (7), 1129–1136. 10.1124/dmd.115.064493 25934574 PMC4468437

[B49] Li M. M. Wang W. P. Wu W. J. Huang M. Yu A. M. (2014). Rapid production of novel pre-MicroRNA agent hsa-mir-27b in *Escherichia coli* using recombinant RNA technology for functional studies in mammalian cells. Drug Metab. Dispos. 42 (11), 1791–1795. 10.1124/dmd.114.060145 25161167 PMC4201134

[B50] Li X. Tian Y. Tu M. J. Ho P. Y. Batra N. Yu A. M. (2019). Bioengineered miR-27b-3p and miR-328-3p modulate drug metabolism and disposition via the regulation of target ADME gene expression. Acta Pharm. Sin. B 9 (3), 639–647. 10.1016/j.apsb.2018.12.002 31193825 PMC6543075

[B51] Liu S. R. da Cunha A. P. Rezende R. M. Cialic R. Wei Z. Y. Bry L. (2016). The host shapes the gut microbiota via fecal MicroRNA. Cell Host Microbe 19 (1), 32–43. 10.1016/j.chom.2015.12.005 26764595 PMC4847146

[B52] Liu S. R. Rezende R. M. Moreira T. G. Tankou S. K. Cox L. M. Wu M. (2019). Oral administration of miR-30d from feces of MS patients suppresses MS-like symptoms in mice by expanding akkermansia muciniphila. Cell Host Microbe 26 (6), 779–794. 10.1016/j.chom.2019.10.008 31784260 PMC6948921

[B53] Mallet J. F. Shahbazi R. Alsadi N. Matar C. (2021). Polyphenol-enriched blueberry preparation controls breast cancer stem cells by targeting FOXO1 and miR-145. Molecules 26 (14), 4330. 10.3390/molecules26144330 34299605 PMC8304479

[B54] Mao L. Zeng Q. C. Su W. J. Song M. L. Li J. C. Xie M. (2021). Elevation of miR-146a inhibits BTG2/BAX expression to ameliorate postoperative cognitive dysfunction following probiotics (VSL#3) treatment. Mol. Neurobiol. 58 (7), 3457–3470. 10.1007/s12035-021-02330-z 33725320

[B55] Marc R. F. Nahum S. (2012). The mechanics of miRNA-mediated gene silencing: a look under the hood of miRISC. Nat. Struct. Mol. Biol. 19 (6), 586586–593593. 10.1038/nsmb.2296 22664986

[B56] Marmol I. Sanchez-De-Diego C. Dieste A. P. Cerrada E. Yoldi M. J. R. (2017). Colorectal carcinoma: A general Overview and future Perspectives in colorectal cancer. Int. J. Mol. Sci. 18 (1), E197. 10.3390/ijms18010197 28106826 PMC5297828

[B57] Masterson C. H. McCarthy S. D. O'Toole D. Laffey J. G. (2020). The role of cells and their products in respiratory drug delivery: the past, present, and future. Expert Opin. Drug Deliv. 17 (12), 1689–1702. 10.1080/17425247.2020.1814732 32842784

[B58] Mathew E. F. Eleni P. George A. M. Georgios P. (2008). Comparison of PubMed, Scopus, Web of science, and google scholar: strengths and weakness. FASEB J. 22, 338. 10.1096/fj.07-9492LSF 17884971

[B59] Mikkel M. Jaslin J. Christian J. Estrid H. Kim H. Vibeke W. (2021). Mucosal microRNAs relate to age and severity of disease in ulcerative colitis. Aging 13, 6359. 10.18632/aging.202715 33647883 PMC7993741

[B60] Miro-Blanch J. Yanes O. (2019). Epigenetic regulation at the interplay between gut microbiota and host metabolism. Front. Genet. 10, 638. 10.3389/fgene.2019.00638 31338107 PMC6628876

[B61] Mody D. Verma V. Rani V. (2021). Modulating host gene expression via gut microbiome-microRNA interplay to treat human diseases. Crit. Rev. Microbiol. 47 (5), 596–611. 10.1080/1040841x.2021.1907739 34407384

[B62] Moloney G. M. Dinan T. G. Clarke G. Cryan J. F. (2019). Microbial regulation of microRNA expression in the brain-gut axis. Curr. Opin. Pharmacol. 48, 120–126. 10.1016/j.coph.2019.08.005 31590111

[B63] Moloney G. M. O'Leary O. F. Salvo-Romero E. Desbonnet L. Shanahan F. Dinan T. G. (2017). Microbial regulation of hippocampal miRNA expression: Implications for transcription of kynurenine pathway enzymes. Behav. Brain Res. 334, 50–54. 10.1016/j.bbr.2017.07.026 28736331

[B64] Moloney G. M. Viola M. F. Hoban A. E. Dinan T. G. Cryan J. F. (2018). Faecal microRNAs: indicators of imbalance at the host-microbe interface? Benef. Microbes 9 (2), 175–183. 10.3920/bm2017.0013 29264965

[B65] Monaghan T. M. Seekatz A. M. Markham N. O. Yau T. O. Hatziapostolou M. Jilani T. (2021). Fecal microbiota transplantation for recurrent clostridioides difficile infection associates with functional alterations in circulating microRNAs. Gastroenterology 161 (1), 255–270.e4. 10.1053/j.gastro.2021.03.050 33844988 PMC8579492

[B66] Naqvi A. R. Fordham J. B. Khan A. Nares S. (2014). MicroRNAs responsive to Aggregatibacter actinomycetemcomitans and Porphyromonas gingivalis LPS modulate expression of genes regulating innate immunity in human macrophages. Innate Immun. 20 (5), 540–551. 10.1177/1753425913501914 24062196 PMC3962801

[B67] Nivedita B. Hui W. Gangduo W. Paul J. B. M Firoze K. (2022). Differential expression of miRNAs in trichloroethene-mediated. Front. Immunol. 13, 868539. 10.3389/fimmu.2022.868539 35422807 PMC9001960

[B68] Pang P. Yu B. Shi Y. C. Deng L. Xu H. C. Wu S. Z. (2018). Alteration of intestinal flora stimulates pulmonary microRNAs to interfere with host antiviral immunity in influenza. Molecules 23 (12), E3151. 10.3390/molecules23123151 30513647 PMC6321108

[B69] Pavithra S. Krisna Moorthi P. Uma Devi R. Ramalingam B. (2021). - monocyte and macrophage miRNA: Potent biomarker and target for host-directed. Front. Immunol. 12, 667206. 10.3389/fimmu.2021.667206 34248945 PMC8267585

[B70] Peruhova M. Peshevska-Sekulovska M. Krastev B. Panayotova G. Georgieva V. Konakchieva R. (2020). What could microRNA expression tell us more about colorectal serrated pathway carcinogenesis? World J. Gastroenterol. 26 (42), 6556–6571. 10.3748/wjg.v26.i42.6556 33268946 PMC7673963

[B71] Peter B. Lali S.-A. Mariannre B.-R. Patrice D. Yamamoto Y. Y. Sieburth L. (2008). Widespread translational inhibition by plant miRNAs and siRNAs. Science 320 (5880), 1185–1190. 10.1126/science.1159151 18483398

[B72] Peter J. T. Ruth E. L. Micah H. Claire F.-L. Rob K. Jeffrey I G. (2007). The human microbiome project. Nature 449 (7164), 804–810. 10.1038/nature06244 17943116 PMC3709439

[B73] Ragusa M. Santagati M. Mirabella F. Lauretta G. Cirnigliaro M. Brex D. (2020). Potential associations among alteration of salivary miRNAs, saliva microbiome structure, and cognitive impairments in autistic children. Int. J. Mol. Sci. 21 (17), E6203. 10.3390/ijms21176203 32867322 PMC7504581

[B74] Rajesha R. Frank J. S. (2017). MicroRNA therapeutics: towards a new era for the management of cancer and other. Nat. Rev. Drug Discov. 16 (3), 203203–222222. 10.1038/nrd.2016.246 28209991

[B75] Rajoka M. S. R. Jin M. L. Zhao H. B. Li Q. Shao D. Y. Huang Q. S. (2018). Impact of dietary compounds on cancer-related gut microbiota and microRNA. Appl. Microbiol. Biotechnol. 102 (10), 4291–4303. 10.1007/s00253-018-8935-3 29589094

[B76] Rashid H. Hossain B. Siddiqua T. Kabir M. Noor Z. Ahmed M. (2020). Fecal MicroRNAs as potential biomarkers for screening and diagnosis of intestinal diseases. Front. Mol. Biosci. 7, 181. 10.3389/fmolb.2020.00181 32850969 PMC7426649

[B77] Rodriguez-Nogales A. Algieri F. Garrido-Mesa J. Vezza T. Utrilla M. P. Chueca N. (2017). Differential intestinal anti-inflammatory effects of Lactobacillus fermentum and Lactobacillus salivarius in DSS mouse colitis: impact on microRNAs expression and microbiota composition. Mol. Nutr. Food Res. 61 (11), 1700144. 10.1002/mnfr.201700144 28752563

[B78] Rodriguez-Nogales A. Algieri F. Garrido-Mesa J. Vezza T. Utrilla M. P. Chueca N. (2018b). Intestinal anti-inflammatory effect of the probiotic Saccharomyces boulardii in DSS-induced colitis in mice: Impact on microRNAs expression and gut microbiota composition. J. Nutr. Biochem. 61, 129–139. 10.1016/j.jnutbio.2018.08.005 30236870

[B79] Rodriguez-Nogales A. Algieri F. Garrido-Mesa J. Vezza T. Utrilla M. P. Chueca N. (2018a). The administration of *Escherichia coli* Nissle 1917 ameliorates development of DSS-induced colitis in mice. Front. Pharmacol. 9, 468. 10.3389/fphar.2018.00468 29867475 PMC5958303

[B80] Sabharwal H. Cichon C. Olschlager T. A. Sonnenborn U. Schmidt M. A. (2016). Interleukin-8, CXCL1, and MicroRNA miR-146a responses to probiotic *Escherichia coli* Nissle 1917 and enteropathogenic *E. coli* in human intestinal epithelial T84 and monocytic THP-1 cells after apical or basolateral infection. Infect. Immun. 84 (9), 2482–2492. 10.1128/iai.00402-16 27297392 PMC4995903

[B81] Santos A. A. Afonso M. B. Ramiro R. S. Pires D. Pimentel M. Castro R. E. (2020). Host miRNA-21 promotes liver dysfunction by targeting small intestinal Lactobacillus in mice. Gut Microbes 12 (1), 1. 10.1080/19490976.2020.1840766 PMC7733982 33300439

[B82] Sarah B. Gyorgy H. (2020). RNA-based therapeutics: From antisense oligonucleotides to miRNAs. Cells 9 (1), 137. 10.3390/cells9010137 PMC7016530 31936122

[B83] Schmidt M. F. (2017). miRNA targeting drugs: The next blockbusters? - Methods Mol. Biol. 1517, 3–22. 10.1007/978-1-4939-6563-2_1 27924471

[B84] Seo M. Anderson G. (2019). Gut-amygdala interactions in autism spectrum disorders: Developmental roles via regulating mitochondria, exosomes, immunity and microRNAs. Curr. Pharm. Des. 25 (41), 4344–4356. 10.2174/1381612825666191105102545 31692435

[B85] Singh R. Zogg H. Ro S. (2021). Role of microRNAs in disorders of gut-brain interactions: Clinical insights and therapeutic alternatives. J. Pers. Med. 11 (10), 1021. 10.3390/jpm11101021 34683162 PMC8541612

[B86] Stanton B. A. (2021). Extracellular vesicles and host-pathogen interactions: A review of inter-kingdom signaling by small noncoding RNA. Genes 12 (7), 1010. 10.3390/genes12071010 34208860 PMC8303656

[B87] Sun Y. L. Sun Y. H. Zhao R. L. (2017). Establishment of MicroRNA delivery system by PP7 bacteriophage-like particles carrying cell-penetrating peptide. J. Biosci. Bioeng. 124 (2), 242–249. 10.1016/j.jbiosc.2017.03.012 28442387

[B88] Tania R. Daniele V. Francesca F. Michale M. Sara B. Francesca P. (2020). Microbiota-derived metabolites in tumor progression and metastasis. Int. J. Mol. Sci. 21 (16), 5786. 10.3390/ijms21165786 PMC7460823 32806665

[B89] TX L. ME R. (2018). MicroRNA. J Allergy Clin. Immunol. 141 (4), 1202–1207. 10.1016/j.jaci.2017.08.034 29074454 PMC5889965

[B90] Veltman K. Hummel S. Cichon C. Sonnenborn U. Schmidt M. A. (2012). Identification of specific miRNAs targeting proteins of the apical junctional complex that simulate the probiotic effect of *E. coli* Nissle 1917 on T84 epithelial cells. Int. J. Biochem. Cell Biol. 44 (2), 341–349. 10.1016/j.biocel.2011.11.006 22101077

[B91] Viggiano D. laniro G. Vanella G. Bibbo S. Bruno G. Simeone G. (2015). Gut barrier in health and disease: focus on childhood. Eur. Rev. Med. Pharmacol. Sci. 19 (6), 10771077–10851085. 25855935

[B92] Wang W. P. Ho P. Y. Chen Q. X. Addepalli B. Limbach P. A. Li M. M. (2015). Bioengineering novel chimeric microRNA-34a for prodrug cancer therapy: High-yield expression and purification, and structural and functional characterization. J. Pharmacol. Exp. Ther. 354 (2), 131–141. 10.1124/jpet.115.225631 26022002 PMC4518075

[B93] Weronika H. Andrea D. L. Krystyna B.-S. (2022). Functional analysis of a frontal miRNA cluster located in the large latency. Viruses 14 (6), 1147. 10.3390/v14061147 35746619 PMC9227234

[B94] Xian-Qian W. Ai-Hua Z. Jian-Hua M. Hui S. Guang-Li Y. Fang-Fang W. (2018). Gut microbiota as important modulator of metabolism in health and disease. RSC Adv. 8 (74), 42380–42389. 10.1039/c8ra08094a 35558413 PMC9092240

[B95] Xiaoran X. Peng Q. Hao W. Peng L. Ju L. Jingshu C. (2022). Circulating exosomal miR-21 mediates HUVEC proliferation and migration through. Ann. Transl. Med. 10 (5), 258. 10.21037/atm-22-475 35402577 PMC8987870

[B96] Xiuqing Z. Jinqing H. Shuhua D. Yaqian T. Chang Q. Ming Z. (2021). Comprehensive bibliometric analysis of the kynurenine pathway in mood disorders. Front. Pharmacol. 12, 687757. 10.3389/fphar.2021.687757 34239441 PMC8258344

[B97] Xue X. C. Feng T. Yao S. X. Wolf K. J. Liu C. G. Liu X. P. (2011). Microbiota downregulates dendritic cell expression of miR-10a, which targets IL-12/IL-23p40. J. Immunol. 187 (11), 5879–5886. 10.4049/jimmunol.1100535 22068236 PMC3226774

[B98] Yang Y. Z. Weng W. H. Peng J. J. Hong L. M. Yang L. Toiyama Y. (2017). Fusobacterium nucleatum increases proliferation of colorectal cancer cells and tumor development in mice by activating toll-like receptor 4 signaling to nuclear factor-kappa B, and up-regulating expression of MicroRNA-21. Gastroenterology 152 (4), 851–866. 10.1053/j.gastro.2016.11.018 27876571 PMC5555435

[B99] Yi D. Y. Kim S. Y. (2021). Human breast milk composition and function in human health: From nutritional components to microbiome and MicroRNAs. Nutrients 13 (9), 3094. 10.3390/nu13093094 34578971 PMC8471419

[B100] Yu A. M. Batra N. Tu M. J. Sweeney C. (2020). Novel approaches for efficient *in vivo* fermentation production of noncoding RNAs. Appl. Microbiol. Biotechnol. 104 (5), 1927–1937. 10.1007/s00253-020-10350-3 31953559 PMC7385725

[B101] Yu A. M. Jian C. Yu A. H. Tu M. J. (2019). RNA therapy: Are we using the right molecules? Pharmacol. Ther. 196, 91–104. 10.1016/j.pharmthera.2018.11.011 30521885 PMC6450780

[B102] Yu S. R. Zhao Z. H. Xu X. Y. Li M. Li P. (2019). Characterization of three different types of extracellular vesicles and their impact on bacterial growth. Food Chem. 272, 372–378. 10.1016/j.foodchem.2018.08.059 30309557

[B103] Yuan C. Burns M. B. Subramanian S. Blekhman R. (2018). Interaction between host MicroRNAs and the gut microbiota in colorectal cancer. Msystems 3 (3), e00205. 10.1128/mSystems.00205-17 29795787 PMC5954203

[B104] Yuan C. Subramanian S. (2019). microRNA-mediated tumor-microbiota metabolic interactions in colorectal cancer. DNA Cell Biol. 38 (4), 281–285. 10.1089/dna.2018.4579 30668143 PMC6477581

[B105] Yun T. Yi R. Mohammed S. Xin H. Chao L. Anil K. (2018). Plant-derived exosomal MicroRNAs shape the gut microbiota. Cell Host Microbe 24 (5), 637–652. 10.1016/j.chom.2018.10.001 30449315 PMC6746408

[B106] Zhang Q. Y. Ho P. Y. Tu M. J. Jilek J. L. Chen Q. X. Zeng S. (2018). Lipidation of polyethylenimine-based polyplex increases serum stability of bioengineered RNAi agents and offers more consistent tumoral gene knockdown *in vivo* . Int. J. Pharm. 547 (1-2), 537–544. 10.1016/j.ijpharm.2018.06.026 29894758 PMC6388695

[B107] Zhang X. S. Yin Y. S. Wang J. C. Battaglia T. Krautkramer K. Li W. V. (2021). Maternal cecal microbiota transfer rescues early-life antibiotic-induced enhancement of type 1 diabetes in mice. Cell Host Microbe 29 (8), 1249–1265. 10.1016/j.chom.2021.06.014 34289377 PMC8370265

[B108] Zhao Y. H. Lukiw W. J. (2018). Bacteroidetes neurotoxins and inflammatory neurodegeneration. Mol. Neurobiol. 55 (12), 9100–9107. 10.1007/s12035-018-1015-y 29637444

[B109] Zhao Y. Y. Tao Q. Y. Li S. Y. Zheng P. Y. Liu J. W. Liang X. (2020). Both endogenous and exogenous miR-139-5p inhibit Fusobacterium nucleatum-related colorectal cancer development. Eur. J. Pharmacol. 888, 173459. 10.1016/j.ejphar.2020.173459 32768506

[B110] Zhu Y. G. Feng X. M. Abbott J. Fang X. H. Hao Q. Monsel A. (2014). Human mesenchymal stem cell Microvesicles for treatment of *Escherichia coli* Endotoxin- induced acute lung injury in mice. Stem Cells 32 (1), 116–125. 10.1002/stem.1504 23939814 PMC3947321

[B111] Zhu Z. X. Huang J. G. Li X. Xing J. Chen Q. Liu R. L. (2020). Gut microbiota regulate tumor metastasis via circRNA/miRNA networks. Gut Microbes 16, 1788891. 10.1080/19490976.2020.1788891 PMC7524358 32686598

